# Bone Morphogenetic Protein 9 Protects against Neonatal Hyperoxia-Induced Impairment of Alveolarization and Pulmonary Inflammation

**DOI:** 10.3389/fphys.2017.00486

**Published:** 2017-07-13

**Authors:** Xueyu Chen, Mar Orriols, Frans J. Walther, El Houari Laghmani, Annemarie M. Hoogeboom, Anne C. B. Hogen-Esch, Pieter S. Hiemstra, Gert Folkerts, Marie-José T. H. Goumans, Peter ten Dijke, Nicholas W. Morrell, Gerry T. M. Wagenaar

**Affiliations:** ^1^Division of Neonatology, Department of Pediatrics, Leiden University Medical Center Leiden, Netherlands; ^2^Department of Molecular Cell Biology, Cancer Genomics Center Netherlands, Leiden University Medical Center Leiden, Netherlands; ^3^Department of Pediatrics, Los Angeles Biomedical Research Institute at Harbor-UCLA Medical Center Torrance, CA, United States; ^4^Department of Pulmonology, Leiden University Medical Center Leiden, Netherlands; ^5^Department of Pharmacology, Utrecht Institute for Pharmaceutical Sciences, Utrecht University Utrecht, Netherlands; ^6^Department of Medicine, University of Cambridge School of Clinical Medicine, Addenbrooke's and Papworth Hospitals Cambridge, United Kingdom

**Keywords:** bronchopulmonary dysplasia, lung fibrosis, activin receptor-like kinase 1, right ventricular hypertrophy, transmembrane protein 100

## Abstract

**Aim:** Effective treatment of premature infants with bronchopulmonary dysplasia (BPD) is lacking. We hypothesize that bone morphogenetic protein 9 (BMP9), a ligand of the TGF-β family that binds to the activin receptor-like kinase 1 (ALK1)-BMP receptor type 2 (BMPR2) receptor complex, may be a novel therapeutic option for BPD. Therefore, we investigated the cardiopulmonary effects of BMP9 in neonatal Wistar rats with hyperoxia-induced BPD.

**Methods:** Directly after birth Wistar rat pups were exposed to 100% oxygen for 10 days. From day 2 rat pups received BMP9 (2.5 μg/kg, twice a day) or 0.9% NaCl by subcutaneous injection. Beneficial effects of BMP9 on aberrant alveolar development, lung inflammation and fibrosis, and right ventricular hypertrophy (RVH) were investigated by morphometric analysis and cytokine production. In addition, differential mRNA expression of *BMP9* and its receptor complex: *ALK1, BMPR2*, and *Endoglin*, and of the ALK1 downstream target *transmembrane protein 100 (TMEM100)* were studied during the development of experimental BPD. Expression of the BMP9 receptor complex and *TMEM100* was studied in human endothelial and epithelial cell cultures and the effect of BMP9 on inflammatory cytokine production and *TMEM100* expression was studied in endothelial cell cultures.

**Results:** *ALK1, ALK2, BMPRII, TMEM100*, and *Endoglin* were differentially expressed in experimental BPD, suggesting a role for BMP9-dependent signaling in the development of (experimental) BPD. TMEM100 was expressed in the wall of blood vessels, showing an elastin-like expression pattern in arterioles. Expression of TMEM100 mRNA and protein was decreased after exposure to hyperoxia. BMP9 treatment of rat pups with hyperoxia-induced experimental BPD reduced alveolar enlargement, lung septal thickness and fibrosis, and prevented inflammation, but did not attenuate vascular remodeling and RVH. The anti-inflammatory effect of BMP9 was confirmed *in vitro*. Highest expression of *ALK1, BMPR2*, and *TMEM100* was observed in human endothelial cell cultures. Stimulation of human endothelial cell cultures with BMP9 reduced their pro-inflammatory cytokine response and induced *TMEM100* expression in pulmonary arterial endothelial cells.

**Conclusion:** BMP9 protects against neonatal hyperoxia-induced BPD by improving aberrant alveolar development, inflammation and fibrosis, demonstrating its therapeutic potential for premature infants with severe BPD.

## Introduction

Experimental and clinical evidence is accumulating that premature birth can have a major impact on neonatal lung development and morbidity that may persist into adulthood (Baraldi and Filippone, 2007). Advanced neonatal intensive care with non-invasive ventilation and surfactant therapy has sharply increased survival of even the youngest premature infants at the expense of increased morbidity, including brain damage due to cerebral bleedings and hypoxia, gastro-intestinal complications, retinopathy, chronic lung disease, and pulmonary hypertension-induced right ventricular hypertrophy (RVH). The immature lung is highly susceptible to injury caused by mechanical ventilation and supplemental oxygen administration during treatment of respiratory distress syndrome (RDS) and this may progress toward chronic lung disease, known as bronchopulmonary dysplasia (BPD). BPD is the most common complication in survivors of very premature birth, born at <30 weeks of gestation (Baraldi and Filippone, 2007). The hallmark of BPD is lung emphysema due to an arrest in alveolar and vascular development, and lung injury. BPD leads to simplification of the alveolar structures and impaired lung function, and is complicated by inflammation and oxidative stress-induced alveolar destruction. Survivors of BPD are severely affected later in life as they end up with persistent impaired lung function and are at high risk of developing respiratory infections with frequent rehospitalizations, asthma, cardiac failure due to pulmonary arterial hypertension (PAH), and possibly chronic obstructive pulmonary disease (COPD) (Baraldi and Filippone, 2007; Wong et al., 2008; Steinhorn, 2010).

Clinical treatment for BPD is mainly supportive as currently available treatment options are limited to caffeine administration to stimulate respiratory drive in the brain, glucocorticoids for weaning from the ventilator and vitamin A administration. However, effective treatment options to prevent or stimulate repair of the injured lung of BPD patients is still lacking. Unfortunately, clinical trials of inhaled nitric oxide (iNO) as treatment option for BPD failed to improve lung function and the translation to the clinic of sildenafil to treat BPD is not an option anymore because the food and drug agency (FDA) issued a safety warning against the use of sildenafil in children after reports of increased mortality rates in children treated for pulmonary hypertension at high concentrations of sildenafil (Kinsella et al., 2014; Perez and Laughon, 2015). Therefore, novel therapeutic drugs need to be identified for the treatment of BPD using suitable animal models, such as new born rodents (Koppel et al., 1994; de Visser et al., 2009, 2010, 2012; Alapati et al., 2011; Hilgendorff et al., 2014; Chen et al., 2016). Newborn rats and mice have immature lungs and develop persistent alveolar simplification, chronic lung inflammation, fibrosis, PAH, and RVH after several days of exposure to hyperoxia, mimicking severe BPD (de Visser et al., 2010, 2012; Madurga et al., 2013; Berger and Bhandari, 2014; Hilgendorff et al., 2014; O'Reilly and Thebaud, 2014). Clinical and experimental evidence is accumulating that excessive transforming growth factor-β (TGF-β)-signaling in premature infants and rodents contributes to the development and severity of lung injury and (experimental) BPD: (1) Increased expression of TGF-β has been observed in the lungs of children with BPD and increased expression of TGF-β and its receptors was found in neonatal and adult animals with lung disease (Lecart et al., 2000; Vicencio et al., 2002; Alejandre-Alcazar et al., 2007, 2008; Morty et al., 2009; Tatler and Jenkins, 2012), (2) Overexpression of TGF-β in neonatal mice and rats resulted in experimental BPD (Gauldie et al., 2003; Vicencio et al., 2004), and (3) TGF-β neutralizing antibodies and pharmacological inhibition of TGF-β reduced lung injury and improved alveolarization in mice with hyperoxia-induced neonatal lung injury (Nakanishi et al., 2007; Sakurai et al., 2011). Interventions in the signaling pathways initiated by the TGF-β family of proteins, including TGF-β and bone morphogenetic proteins (BMPs), may therefore have important therapeutic potential for BPD (Morty et al., 2009). Although, BMP9 protects against PAH in adult mice and rats (Long et al., 2015) and BMP type II receptor (BMPRII) dysfunction enhances lung inflammation (Kim et al., 2013; Talati et al., 2014; Soon et al., 2015), the role of this TGF-β family member in neonatal chronic lung disease is unknown. BMP9 exerts its multiple biological effects after binding to a heterotetrameric transmembrane receptor complex that consists of activin receptor-like kinase 1 (ALK1) or ALK2 with ALK1 being the high affinity receptor for BMP9, and BMPRII, which may be enhanced by the accessory receptor endoglin (Upton et al., 2009; Pardali and Ten Dijke, 2012). Activated ALK1 (or ALK2) phosphorylates Smad1/5/8, which form complexes with Smad4 that translocate to the nucleus to regulate gene expression by interacting with other transcription factors. Alternatively, ALK1/BMPRII signaling may be mediated via non-Smad signaling, e.g., by activating MAP kinases (Pardali and Ten Dijke, 2012). Indirect evidence for a role of BMP9-ALK1-dependent signaling comes from our previous studies on identifying novel genes in the pathogenesis of BPD using a DNA array experimental setup (Wagenaar et al., 2004) in which we identified the unknown expressed sequence tags with accession numbers AI168935 and AI058357, as being transmembrane protein 100 (TMEM100), a downstream target gene of ALK1 (Somekawa et al., 2012), with a very high differential expression in rat pups with experimental BPD.

The effect of BMP9 on the clinical progression of BPD is unknown. To advance our knowledge on BMP9 signaling in neonatal cardiopulmonary disease, we studied (1) the spatial and/or temporal expression of BMP9, its receptor complex and its downstream target transmembrane protein 100 (TMEM100; Somekawa et al., 2012) in neonatal rat lung, (2) the beneficial effects of BMP9 in neonatal rats with hyperoxia-induced BPD and investigated lung alveolarization, inflammation, fibrosis, vascular remodeling, and RVH as described previously (de Visser et al., 2010; Chen et al., 2016), and (3) BMP9 signaling in cultured human epithelial and endothelial cells and the anti-inflammatory potential of BMP9 in cultured human endothelial cells.

## Materials and methods

### Animals

All animal experiments were performed in accordance with the Institute for Laboratory Animal Research Guide for the Care and Use of Laboratory Animals and were approved by the Animal Ethical Committee (Leiden University Medical Center, Leiden, the Netherlands). Six months old adult Wistar rats (*N* = 6) were anesthetized with an intraperitoneal injection of ketamine (50 mg/kg) and xylazine (50 mg/kg) and exsanguinated by cutting the abdominal blood vessels. Organs were stored at −80°C until isolation of RNA for real time RT-PCR. For each intervention experiment, newborn Wistar rat pups from 3 to 5 litters were pooled and assigned ad random to 4 experimental groups: an oxygen-NaCl group (*N* = 6), an oxygen-BMP9 group (*N* = 6), and two room air (RA)-exposed control groups (*N* = 6 each). All oxygen-exposed pups were housed together in Plexiglas chambers and were exposed continuously to 100% oxygen for 10 days. Pups were fed by foster dams and received twice a day subcutaneous injections with either 2.5 μg/kg of BMP9 dissolved in 100 μl 0.9% NaCl or solvent only from day 2 after birth until day 10. Recombinant BMP9 consisting of the human growth factor domain and the mouse prodomain was obtained from Pfizer, as previously described (Long et al., 2015). To avoid oxygen toxicity foster dams were rotated daily: 24 h in hyperoxia and 48 h in RA. Once a day evidence of disease, mortality, body weight, and oxygen concentration, were recorded. On day 10 rat pups were exsanguinated under ketamine and xylazine anesthesia. Hereafter, lungs and hearts were collected. Lungs were fixed under constant pressure (27 cm H_2_O) in formalin for histology studies or snap-frozen in liquid nitrogen for fibrin deposition assay, cytokine assays and RT-qPCR as described previously (de Visser et al., 2009, 2010). Separate experiments were performed to obtain: (1) formalin fixed lung and heart tissue for histology (*N* = 8); (2) lung homogenates for fibrin deposition (*N* = 8); (3) broncho-alveolar lavage fluid (BALF) for protein measurements (*N* = 10); (4) lung tissue from neonates with experimental BPD and RA controls on days 1, 3, 6, and 10 after birth for RT-PCR (*N* = 6–8). For all parameters, at least two independent experiments were performed.

### Histology

Lung and heart tissue was fixed in formalin and embedded in paraffin. Four micrometers thick paraffin-embedded tissue sections were deparaffinized and subsequently stained with hematoxylin and eosin (HE). In addition, lung tissue sections were immune-stained with anti-ED-1 (monocytes and macrophages; 1:5), anti-myeloperoxidase (MPO, RB-373-A1, Thermo Fisher Scientific, Fremont, CA, USA; diluted 1:1,500), anti-α smooth muscle actin (αSMA, A2547, Sigma-Aldrich, St. Louis, MO, USA; diluted 1:20,000), anti-von Willebrand factor (vWF, A0082, Dako Cytomation, Glostrup, Denmark; diluted 1:4,000), anti-collagen III (COL3A1, ab7778; Abcam; diluted 1:3,000), anti-pSMAD1 [diluted 1:2,000; this antibody cross-reacts with pSMAD5 and pSMAD8, which also act downstream of BMP type I receptors (Persson et al., 1998; Rosendahl et al., 2002)], anti-pSMAD2 (diluted 1:2,000), (Persson et al., 1998; Rosendahl et al., 2001) and anti-transmembrane protein 100 (TMEM100, GTX83508; Gene Tex, Irvine, CA, USA; diluted 1:400), using the chromogenic substrate NovaRed or NovaRed and Vector SG Substrate on αSMA and vWF double stained sections, respectively (Vector, Burlingame, CA, USA), and counterstained briefly with hematoxylin using standard methods (de Visser et al., 2009, 2010). Furthermore, elastin was visualized on Hart's stained lung sections (Simon et al., 2010). We used a Weibel type II ocular micrometer (Olympus, Zoeterwoude, The Netherlands) for morphometric analysis of the lung, (Wagenaar et al., 2004). Different (immuno)histochemically stained lung sections were used for each quantification. However, alveolar crests and pulmonary arteriolar wall thickness were determined on the same αSMA stained section. To exclude potential effects of heterogeneous alveolar development we investigated alveolar enlargement in experimental BPD in two different ways by studying mean linear intercept (MLI) and the number of alveolar crests. The MLI, determined on hematoxylin and eosin stained lung sections, was assessed in 10 non-overlapping fields at a 200x magnification for each animal (Dunnill, 1962; de Visser et al., 2010). The quantification of alveolar crests is an indicator of the number of alveoli (Yi et al., 2004). The number of alveolar crests (Yi et al., 2004) was determined on αSMA-stained lung sections at a 400x magnification in 10 non-overlapping fields for each animal and were normalized to tissue and field. The number of MPO-positive neutrophilic granulocytes or ED-1 positive monocytes and macrophages was determined in the alveolar compartment and normalized to field and expressed as cells per mm^2^. Twenty fields in one section were studied at a 400x magnification for each experimental rat. HE-stained lung sections were used at a 400x magnification for alveolar septal thickness measurements by averaging 100 measurements per 10 representative fields. The number of vessels per field were counted at a 200x magnification in vWF-stained lung sections to determine capillary density. At least 10 representative fields per experimental animal were investigated. Results were expressed as relative number of vessels per mm^2^. Arteriolar wall thickness was assessed twice in elastin- or αSMA-stained lung sections at a 1,000x magnification by averaging at least 10 vessels with a diameter of <30 μm per rat for each of the two different staining methods. Medial wall thickness was calculated from the formula “percent wall thickness = $\frac{2*wall\cdot thickness}{external\cdot diameter}*$100” (Koppel et al., 1994). Muscularization of small arterioles (<50 μm) was determined on αSMA and vWF double stained lung sections, using the 50% αSMA layer circumference as a cutoff at a 400x magnification by counting 50 blood vessels per lung section (Alapati et al., 2011; Chen et al., 2011). We excluded fields with large blood vessels or bronchioli from the analysis. Right and left ventricular free wall thickness was assessed at a 40x magnification in a transversal HE-stained section taken halfway the long axis by averaging 6 measurements per structure. For each heart RVH was calculated by dividing average RV free wall thickness and average LV free wall thickness. For morphometric studies in lung and heart, 6–8 and 10 rat pups per experimental group were studied, respectively. NIH Image J software was used for quantitative morphometry. Two independent researchers blinded to the treatment strategy performed the analysis (Yi et al., 2004; de Visser et al., 2010; Chen et al., 2016).

### Fibrin, IL6, and MCP1 detection assays

Extravascular fibrin deposition was quantified in lung tissue homogenates by Western blotting, using rat fibrin as a standard by infrared detection (Odyssey infrared imaging system, Licor Biosciences, Lincoln, NE, USA) as described previously (de Visser et al., 2009, 2010; Wagenaar et al., 2013). IL6 and MCP1 were quantified in lung homogenates by ELISA, essentially as recommended by the manufacturer (IL6: RRF600CKX and MCP1: RRF423CKC, Antigenix America Inc., NY, USA).

### Bronchoalveolar lavages and protein assay

Lung lavages and total protein assay were performed as previously described (de Visser et al., 2009; Wagenaar et al., 2013).

### Real-time RT-qPCR

RT-qPCR was performed on a Light Cycler 480 (Roche, Almere, The Netherlands) at the Leiden Genome Technology Center (Leiden, The Netherlands), using first-strand cDNA synthesized from total RNA [SuperScript Choice System (Life Technologies, Breda, the Netherlands)] and β-actin as a housekeeping gene reference. RNA was isolated from lung tissue homogenates (RNA-Bee, Tel-Test Inc, Bio-Connect BV, Huissen, the Netherlands), as described previously (Wagenaar et al., 2004, 2013). Primers are listed in Table 1.

**Table 1:** Sequences of oligonucleotides for forward and reverse primers for real-time RT-PCR.

**Gene**	**Species**	**Forward primer**	**Reverse primer**
*ALK1*	Rat	5′-CGTGCTGGTCAAGAGCAACTT-3′	5′-GCTTTGCGAGTGCATCACA-3′
*ALK2*	Rat	5′-GGAAGTGGCCAGGAGGAT-3′	5′-GGGTCATTGGGAACAACATC-3′
*BMP9*	Rat	5′-AAGGGACCAGTTGCCATTC-3′	5′-CAGACCCATATACCCCAGTCA-3′
*BMPRII*	Rat	5′-CCCCGAGGAGATCATTACAA-3′	5′-ACGTGCCACCATTCTTTACC-3′
*Endoglin*	Rat	5′-GCTGCGGCATGAAAGTGA-3′	5′-GGTAAGCCTGATGGCAAATTG-3′
*IL6*	Rat	5′-ATATGTTCTCAGGGAGATCTTGGAA-3′	5′-TGCATCATCGCTGTTCATACAA-3′
*TMEM100*	Rat	5′-TGGTCCTTCTCTCCCAAGTCA-3′	5′-CAGGGTGGAAGCTCACAAAAA-3′
β-*actin*	Rat	5′-TTCAACACCCCAGCCATGT-3′	5′-AGTGGTACGACCAGAGGCATACA-3′
*ALK1*	Human	5′-CTGGTTCCGGGAGACTGAGAT-3′	5′-TGCGGGAGGTCATGTCTGA-3′
*ALK2*	Human	5′-TGCCTTCGAATAGTGCTGTC-3′	5′-CATCAAGCTGATTGGTGCTC-3′
*ARP*	Human	5′-CACCATTGAAATCCTGAGTGATGT-3′	5′-TGACCAGCCGAAAGGAGAAG-3′
*BMPRII*	Human	5′-AACTGTTGAGCTGATTGGC-3′	5′-CGGTTTGCAAAGGAAACAC-3′
*ID1*	Human	5′-CTGCTCTACGACATGAACGG-3′	5′-GAAGGTCCCTGATGTAGTCGAT-3′
*IL6*	Human	5′-CCCACACAGACAGCCACTCA-3′	5′-CCGTCGAGGATGTACCGAAT-3′
*MCP1*	Human	5′-GCAATCAATGCCCCAGTCA-3′	5′-GCCTCTGCACTGAGATCTTCCT-3′
*TMEM100*	Human	5′-TGCTGTGGTTGTCTTCATCG-3′	5′-CTCTCCCGTCTCTTGGCTTTC-3′

### Cell culture experiments

Human pulmonary artery endothelial cells (PAECs; Lonza) were maintained in EGM-2 with 10% fetal bovine serum (FBS) and were used for experiments between passages 4 and 8. The Human microvascular endothelial cell line HMEC-1 was cultured in MDCB131 (Invitrogen) containing 10% FBS, 10 mM L-glutamine (Sigma), 1 μg/ml hydrocortisone (Sigma), and 10 ng/ml human epidermal growth factor (Sigma). Both endothelial cell sources were grown on tissue culture plates coated with 0.1% gelatin at 37°C in 5% CO_2_. Primary bronchial epithelial cells (PBEC) were obtained from anonymized tumor-free lung tissue obtained from lung cancer patients at lung resection surgery by enzymatic digestion as described previously (van Wetering et al., 2000). Cells were cultured submerged in a 1:1 mixture of DMEM (Gibco, Grand Island, NY) and bronchial epithelial growth medium (BEGM; Clonetics, San Diego, CA) supplemented with 0.4% (w/v) bovine pituitary extract (BPE), 0.5 ng/ml epidermal growth factor (EGF), 5 μg/ml insulin, 0.1 ng/ml retinoic acid, 10 μg/ml transferrin, 1 μM hydrocortisone, 6.5 ng/ml T3, 0.5 μg/ml epinephrine (all from Clonetics), 1.5 μg/ml bovine serum albumin (BSA; Sigma), 1 mM HEPES (Gibco), 100 U/ml penicillin, and 100 μg/ml streptomycin (Bio Whittaker, Walkersville, MD; van Wetering et al., 2007). Human pulmonary mucoepidermoid carcinoma cell line NCI-H292 was cultured in RPMI 1640 (Invitrogen) containing 10% FBS. All cells were grown in a humidified 5% CO_2_ water-jacketed incubator at 37°C and routinely tested for absence of mycoplasma contamination. At confluence, cells were starved overnight (0% FBS or growth factor) and incubated with recombinant human BMP9 (5 ng/ml; 3209-BP/CF, R&D Systems, Minneapolis, MN) for 4 h. A dose of 5 ng/ml of BMP9 was chosen based on effective stimulation of BMP9/ALK1/pSMAD-dependent signaling with 5 ng/ml of BMP9 in pulmonary arterial endothelial cells in the context of pulmonary arterial hypertension (Long et al., 2015) and the culture times in the presence of BMP9 where chosen based on previous studies on inflammatory cytokine production by endothelial cells (Nilsen et al., 1998; Gargalovic et al., 2006). Thereafter, RNA was extracted and quantitative PCR was used to determine mRNA expression. Total RNA was isolated with the NucleoSpin RNA II kit (BIOKE, Leiden, Netherlands) according to the supplier's manual. cDNA was synthesized with the RevertAid First Strand cDNA Synthesis Kit (Thermo Scientific, Leusden, Netherlands). Real-time quantitative PCR was performed on a CFX connect real-time PCR system (Bio-Rad, Veenendaal, Netherlands). All data were analyzed in triplicate using acidic ribosomal protein (ARP) as a gene reference and the non-stimulated condition was set to 1. Relative expression levels are presented as mean ± SEM. Primers are listed in Table 1.

### Iodinated BMP9 binding assay

Iodination of BMP9 was performed according to the chloramine T method and cells were subsequently affinity labeled with the radioactive ligand as described before (Scharpfenecker et al., 2007). Cell lysates were immunoprecipiated with specific antibodies against ALK1, ALK2, ALK5, Endoglin, and BMPR2. The generation and characterization of these antibodies have been previously published (ten Dijke et al., 1994; Yamashita et al., 1994; Rosenzweig et al., 1995).

### Statistical analysis

Values are expressed as mean ± SEM. Differences between experimental groups in the *in vivo* experiments were analyzed by one-way ANOVA, followed by Tukey's multiple comparisons test. For comparison of survival curves, Kaplan–Meier analysis followed by a log rank test was performed. Differences between groups in the *in vitro* experiments were analyzed by Student *t-*test. For statistical analysis the GraphPad Prism version 7 software package was used (San Diego, CA, USA). A *p* < 0.05 was considered statistically significant.

## Results

### Expression of genes involved in BMP9-dependent signaling

**Figure 1:**
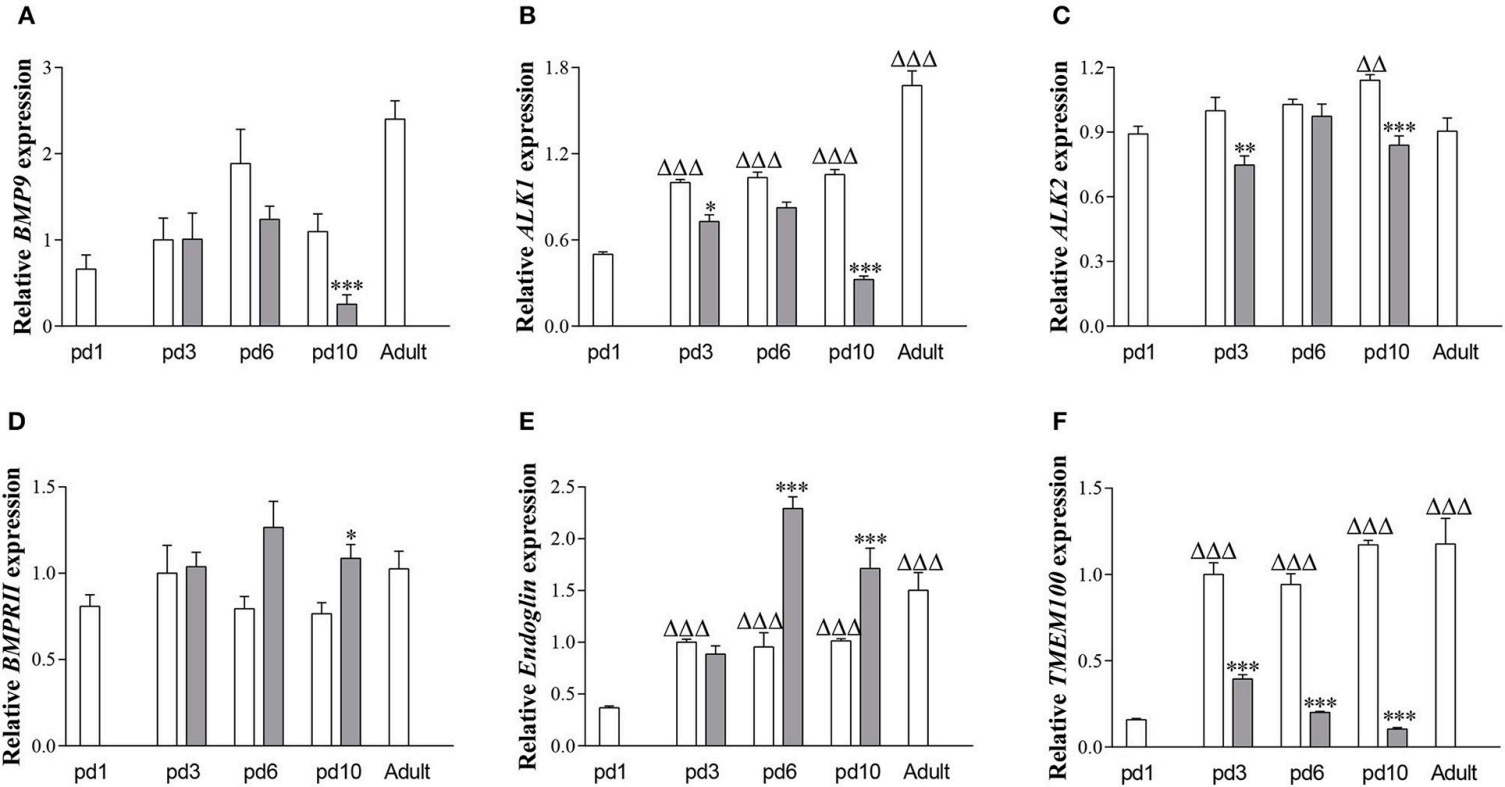
Relative mRNA expression of bone morphogenetic protein 9 (BMP9; **A**) activin receptor-like kinase 1 (ALK1; **B**) activin receptor-like kinase 2 (ALK2; **C**) BMP type II receptor (BMPRII; **D**) Endoglin **(E)** and Transmembrane protein 100 (TMEM100; **F**) in lung of rat pups on days 1, 3, 6, and 10 after birth and in adult rats during normal development in RA (white bars) and after exposure to 100% O_2_ (shaded bars), using β-actin as a house keeping gene reference. Values are expressed as mean ± SEM (*N* = 6–10). Differences between groups were analyzed by one-way ANOVA, followed by Tukey's multiple comparisons test. ^*^p < 0.05, ^**^p < 0.01, and ^***^p < 0.001 vs. own age-matched RA controls. ^ΔΔ^*p* < 0.01 and ^ΔΔΔ^*p* < 0.001 vs. RA control on day 1.

In RA-exposed rat lungs (Figure 1), mRNA expression of *ALK1* (Figure 1B; *p* < 0.001), Endoglin (Figure 1E; *p* < 0.001), and *TMEM100* (Figure 1F; *p* < 0.001) increased with advancing age, whereas mRNA expression of *BMP9* (Figure 1A), *ALK2* (Figure 1C), and *BMPRII* (Figure 1D) did not change significantly. After exposure to hyperoxia for 10 days, mRNA expression of *BMP9* (*p* < 0.001), *ALK1* (*p* < 0.001), *ALK2* (*p* < 0.001), and *TMEM100* (*p* < 0.001) was lower and that of *BMPRII* (*p* < 0.05) and *Endoglin* (*p* < 0.001) was significantly higher than in RA controls.

### Expression of TMEM100 in neonatal lung

**Figure 2:**
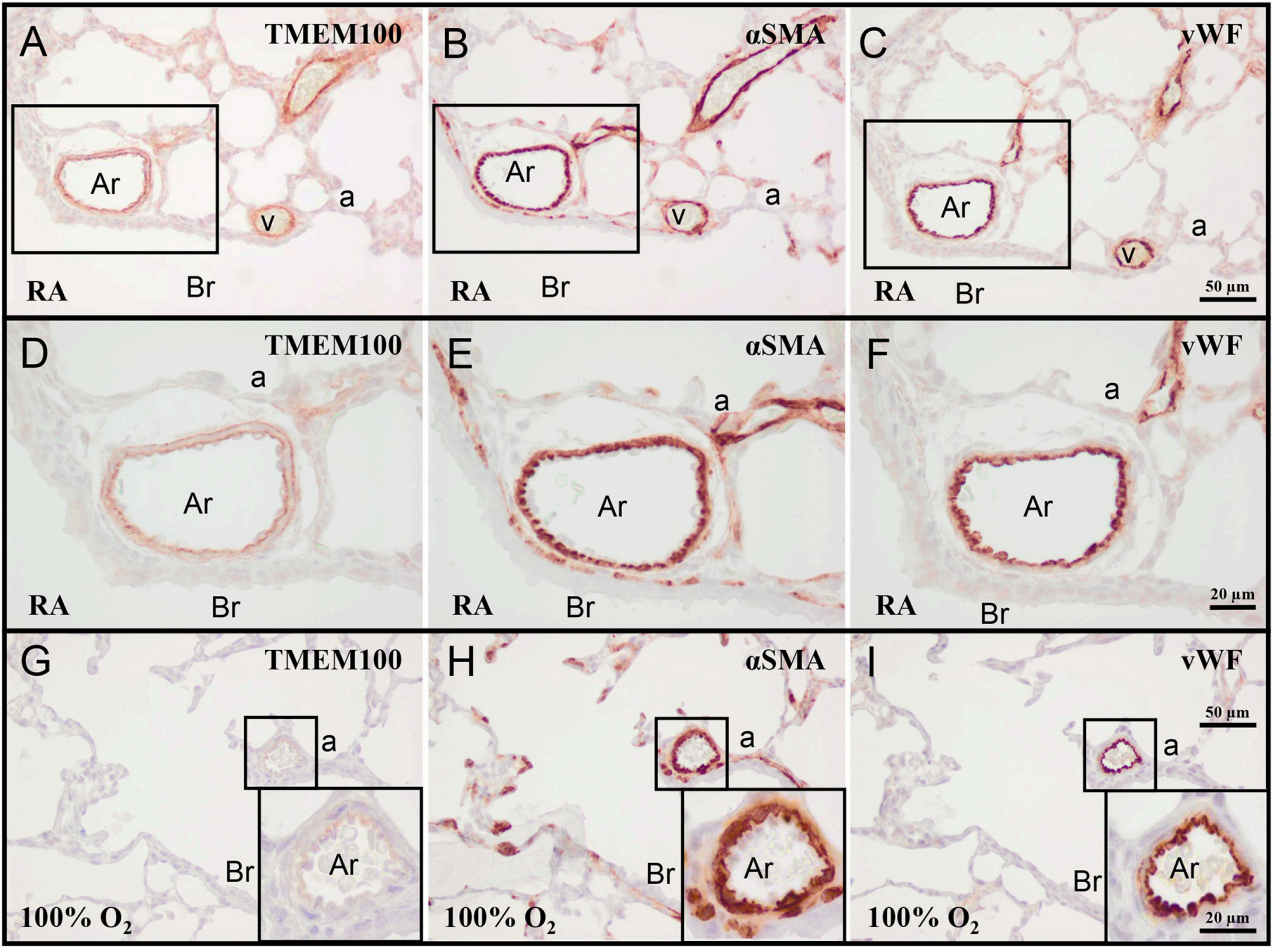
Representative lung sections stained for transmembrane protein 100 (TMEM100; **A,D,G**), α smooth muscle actin (αSMA; **B,E,H**), and von Willebrand Factor (vWF; **C,F,I**) of rat pups kept in RA **(A–F)** or 100% O_2_ **(G–I)** until 10 days of age. Boxed areas in panels **(A–C)** are shown as a high power magnification in panels **(D–F)**, respectively. The boxed areas in panels **(G–I)** are shown in the inset in the right lower boxed corner as a high power magnification. a, alveolus; Ar, arteriole; Br, bronchus; v, vein.

On day 10 TMEM100 was expressed in the vessel wall of veins and arterioles (Figures 2A–C). In arterioles, TMEM100 protein expression (Figure 2D) had a staining pattern very similar to elastin (see **Figure 7E**), showing a double layer, in which the internal elastic lamina is localized directly under the vWF-positive endothelial layer (Figures 2E,F). Exposure to hyperoxia resulted in a decrease in TMEM100 expression in pulmonary arterioles and veins. In very severe BPD TMEM100 protein levels were very low and only expressed in the wall of small arterioles (Figures 2G–I).

### Expression of pSMAD1 and pSMAD2 in neonatal lung

**Figure 3:**
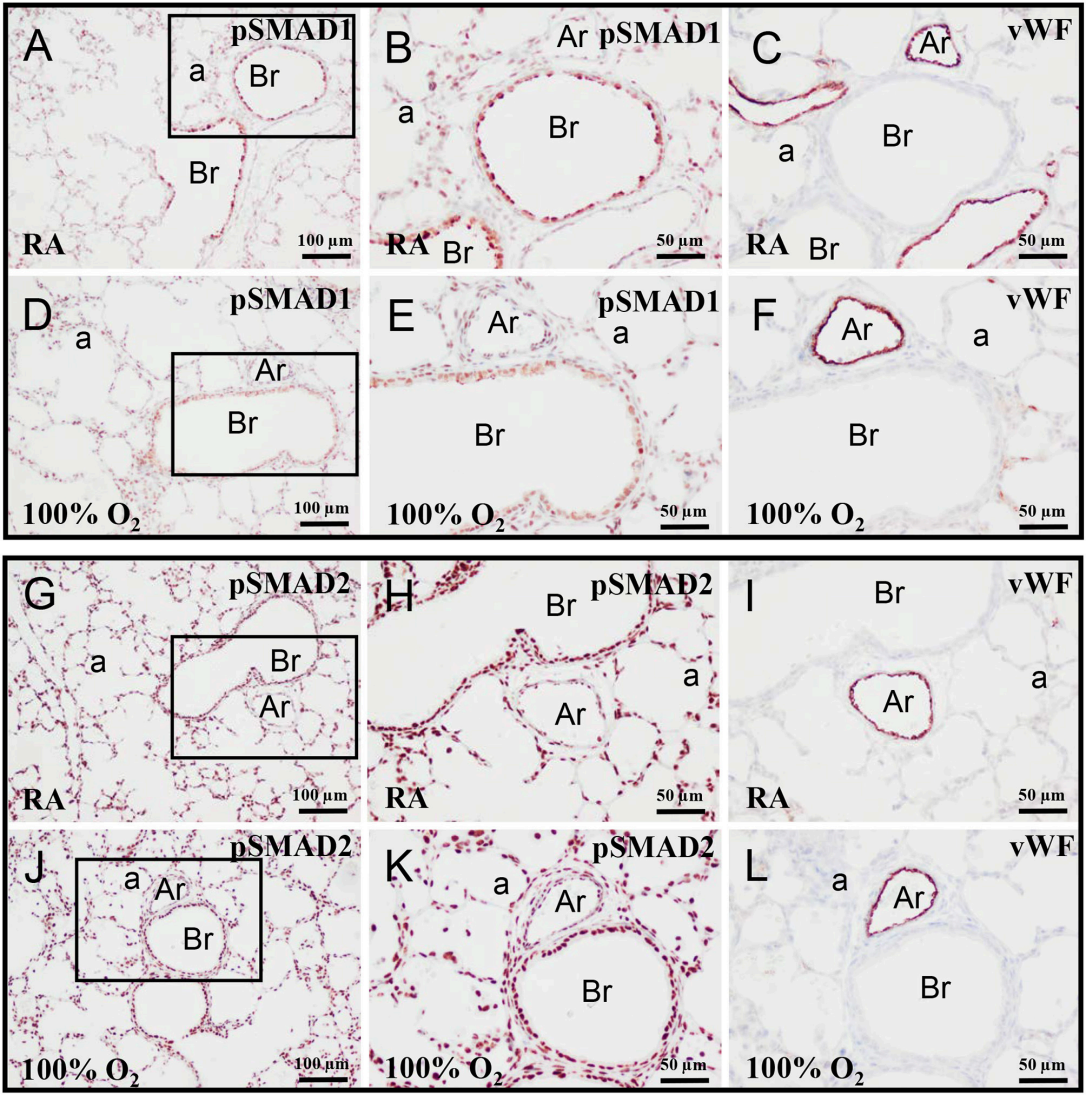
Representative lung sections stained for pSMAD1 **(A,B,D,E)**, pSMAD2 **(G,H,J,K)**, and von Willebrand Factor (vWF; **C,F,I,L**) of rat pups kept in RA **(A–C,G–I)** or 100% O_2_ **(D–F,J–L)** until 10 days of age. The boxed areas in panels **(A,D,G,J)** are shown as high power magnification in panels **(B,E,H,K)**, respectively. a, alveolus; Ar, arteriole; Br, bronchus.

On day 10 pSMAD1 (Figures 3A,B,D,E) and pSMAD2 (Figures 3G,H,J,K) were expressed in multiple cell types, including vascular endothelial cells (Figures 3C,F,I,L), and showed, as expected, a nuclear staining pattern, because both activated SMADs are directed toward the nucleus after binding to SMAD4 (Pardali and Ten Dijke, 2012). Exposure to hyperoxia had no effect on this staining pattern.

### Effects of BMP9 on growth and survival

**Figure 4:**
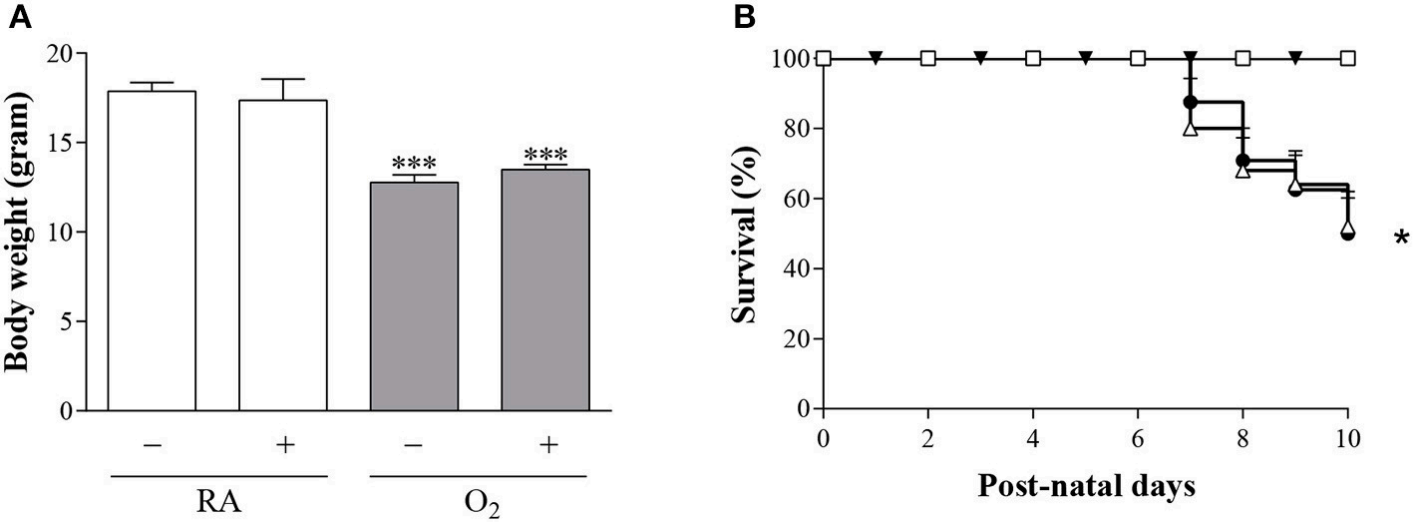
Body weight **(A)** in RA (white bars) and O_2_-exposed (100% O_2_ for 10 days; shaded bars) rat pups injected with 0.9% NaCl or BMP9 (2.5 μg kg^−1^ twice daily for 10 days). Data are expressed as mean ± SEM. Differences between groups were analyzed by one-way ANOVA, followed by Tukey's multiple comparisons test. ^***^p < 0.001 vs. age-matched 0.9% NaCl treated RA controls. Kaplan-Meier survival curves **(B)** of BMP9-treated rat pups exposed to 100% O_2_ (Δ), age-matched O_2_-exposed controls (•) and room air exposed controls treated with BMP9 (□) or 0.9% NaCl (▾) for 10 days after birth. Data are expressed as mean percentage ± SEM of pups surviving at the observed time point. *N* = 18–24. ^*^p < 0.05 vs. age-matched 0.9% NaCl treated RA controls.

Body weight after 10 days in RA (Figure 4A) was comparable in BMP9 and NaCl-treated Wistar rat pups (18 g) and decreased significantly to 13 g after exposure to hyperoxia for 10 days (*p* < 0.001), compared to RA controls. All RA pups survived (Figure 4B) after treatment with either 0.9% NaCl or BMP9. Exposure to 100% O_2_ for 10 days resulted in a 46% drop in survival (*p* < 0.05), compared to RA controls and was not influenced by the administration of BMP9 (Figure 4B).

### Effects of BMP9 on lung airway development, inflammation, and collagen deposition

**Figure 5:**
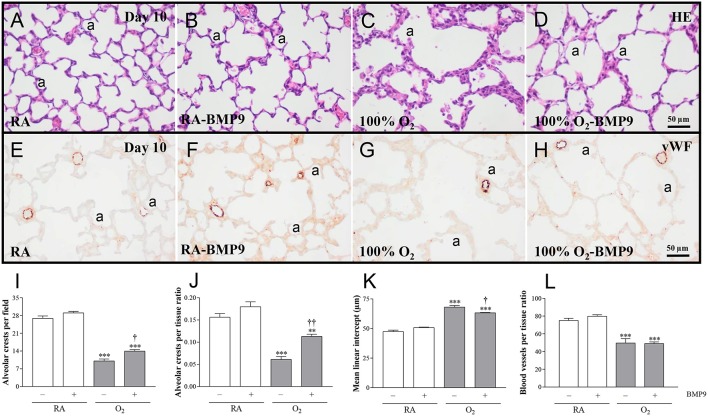
Representative HE-stained lung sections **(A–D)** and lung sections stained for von Willebrand Factor (vWF; **E–H**) in rat pups kept in RA **(A,B,E,F)** or 100% O_2_ **(C,D,G,H)** until 10 days of age. Lung morphometry, including the quantifications of alveolar crests **(I,J)**, mean linear intercept **(K)** and number of pulmonary vessels per tissue ratio **(L)** was determined on paraffin sections in Wistar rats on day 10 kept in RA (open bars) or 100% O_2_ for 10 days (shaded bars). Pups were injected daily with 0.9% NaCl or BMP9 (2.5 μg kg^−1^ twice daily for 10 days). Values are expressed as mean ± SEM. *N* = 8. Differences between groups were analyzed by one-way ANOVA, followed by Tukey's multiple comparisons test. ^**^p < 0.01 and ^***^p < 0.001 vs. RA controls. ^†^*p* < 0.05 and ^††^*p* < 0.01 vs. age-matched O_2_-exposed controls. Three independent experiments were performed. a, alveolus.

**Figure 6:**
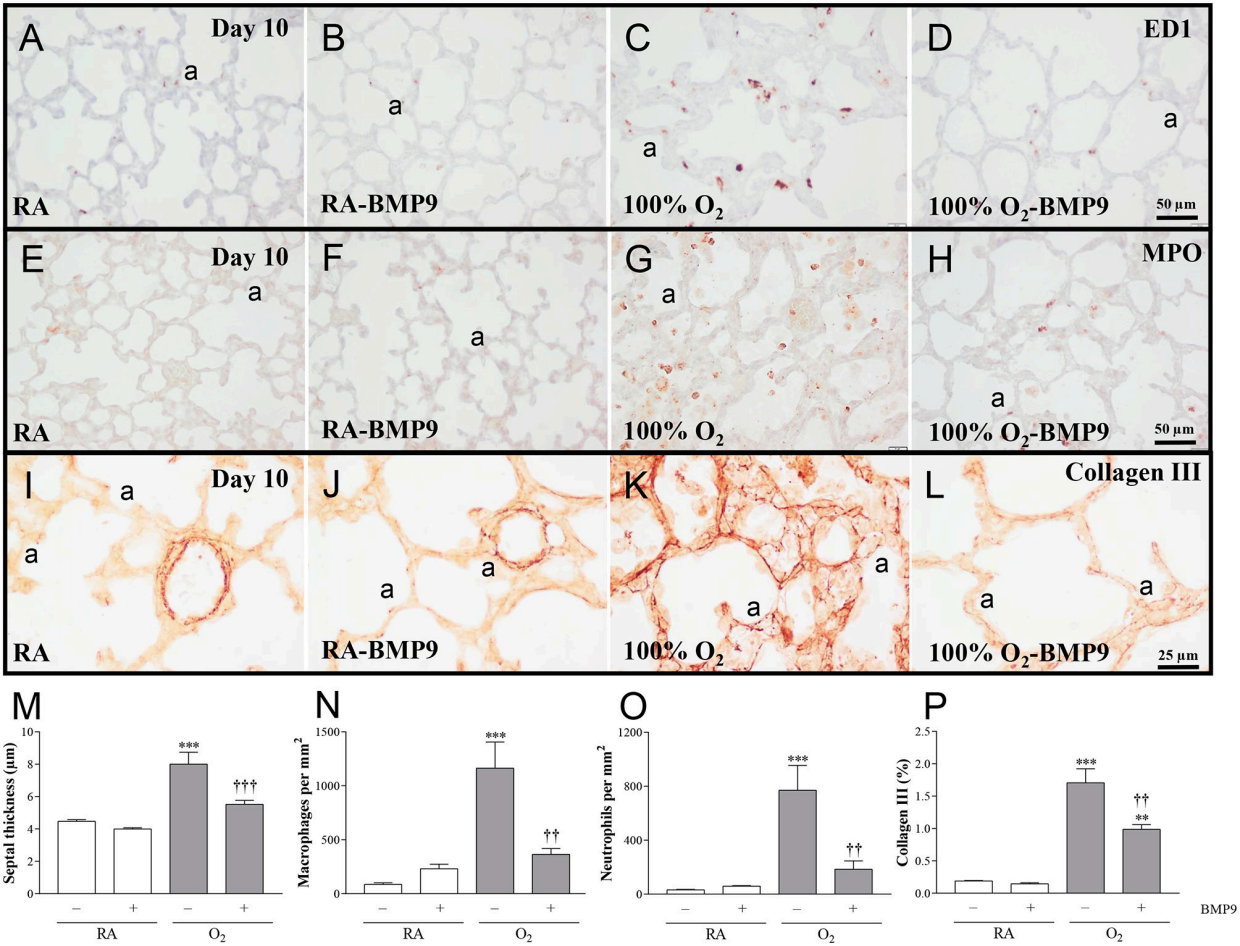
Representative lung sections stained for the macrophage marker ED1 **(A–D)**, neutrophilic granulocyte marker MPO **(E–H)**, or Collagen III **(I–L)** in rat pups kept in room-air (RA; **A,B,E,F,I,J**) or 100% O_2_ **(C,D,G,H,K, L)** until 10 days of age. Quantification of septal thickness **(M)** the pulmonary influx of macrophages **(N)** and neutrophilic granulocytes **(O)**, and collagen III deposition **(P)** was determined on lung paraffin sections in Wistar rats after exposure to RA (open bars) or 100% O_2_ for 10 days (shaded bars). Pups were injected subcutaneously daily with 0.9% NaCl or BMP9 (2.5 μg kg^−1^ twice daily for 10 days). Values are expressed as mean ± SEM. *N* = 8. Differences between groups were analyzed by one-way ANOVA, followed by Tukey's multiple comparisons test. ^**^p < 0.01 and ^***^p < 0.001 vs. RA controls. ^††^*p* < 0.01 and ^†††^*p* < 0.001 vs. age-matched O_2_-exposed controls. Three independent experiments were performed. a, alveolus.

Administration of BMP9 during normal neonatal development for 10 days did not have negative effects on the lung regarding the number of alveolar crests, mean linear intercept (MLI), pulmonary vessel density, alveolar septal thickness, influx of macrophages and neutrophilic granulocytes, and collagen IIIA expression (Figures 5, 6). Exposure to 100% O_2_ for 10 days resulted in a heterogeneous distribution of enlarged alveoli, which was demonstrated by a reduced number of alveolar crests [2.6-fold, *p* < 0.001; Figures 5C,I (per field) and Figure 5J (per tissue ratio)] and increased MLI (1.4-fold, *p* < 0.001; Figure 5K) compared to RA controls. These simplified alveoli were surrounded by thick septa (1.8-fold, *p* < 0.001; Figures 5C, 6M). In addition, exposure to hyperoxia reduced the number of blood vessels per tissue ratio (1.5-fold, *p* < 0.001; Figure 5L) compared to RA-exposed controls. Furthermore, neonatal exposure to 100% O_2_ induced an inflammatory and fibrotic response, characterized by a significant pulmonary influx of macrophages (13.5-fold, *p* < 0.001; Figures 6C,N) and neutrophils (24.8-fold, *p* < 0.001; Figures 6G,O), and increased collagen IIIA deposition in thick alveolar septa (8.9-fold, *p* < 0.001; Figures 6K,P), compared to RA controls. Treatment of hyperoxia-induced experimental BPD with BMP9 for 10 days improved aberrant alveolar development by increasing the number of alveolar crests [1.4-fold, *p* < 0.05; Figures 5D,I (per field) and 1.8-fold, *p* < 0.01; Figures 5D,J (per tissue ratio)] and reducing MLI (1.1-fold, *p* < 0.05; Figure 5K), and decreasing alveolar septal thickness (1.5-fold, *p* < 0.001; Figures 5D, 6M). Furthermore, BMP9 attenuated the hyperoxia-induced inflammatory and fibrotic response by preventing the influx of macrophages (3.2-fold, *p* < 0.01; Figures 6D,N) and neutrophils (4.2-fold, *p* < 0.01; Figures 6H,O), and reducing collagen IIIA expression (1.7-fold, *p* < 0.01; Figures 6L,P), respectively, compared to O_2_-exposed controls. However, beneficial effects on reduced hyperoxia-induced vascularization (Figure 5L) were absent.

### Effects of BMP9 on pulmonary vascular remodeling and right ventricular hypertrophy

**Figure 7:**
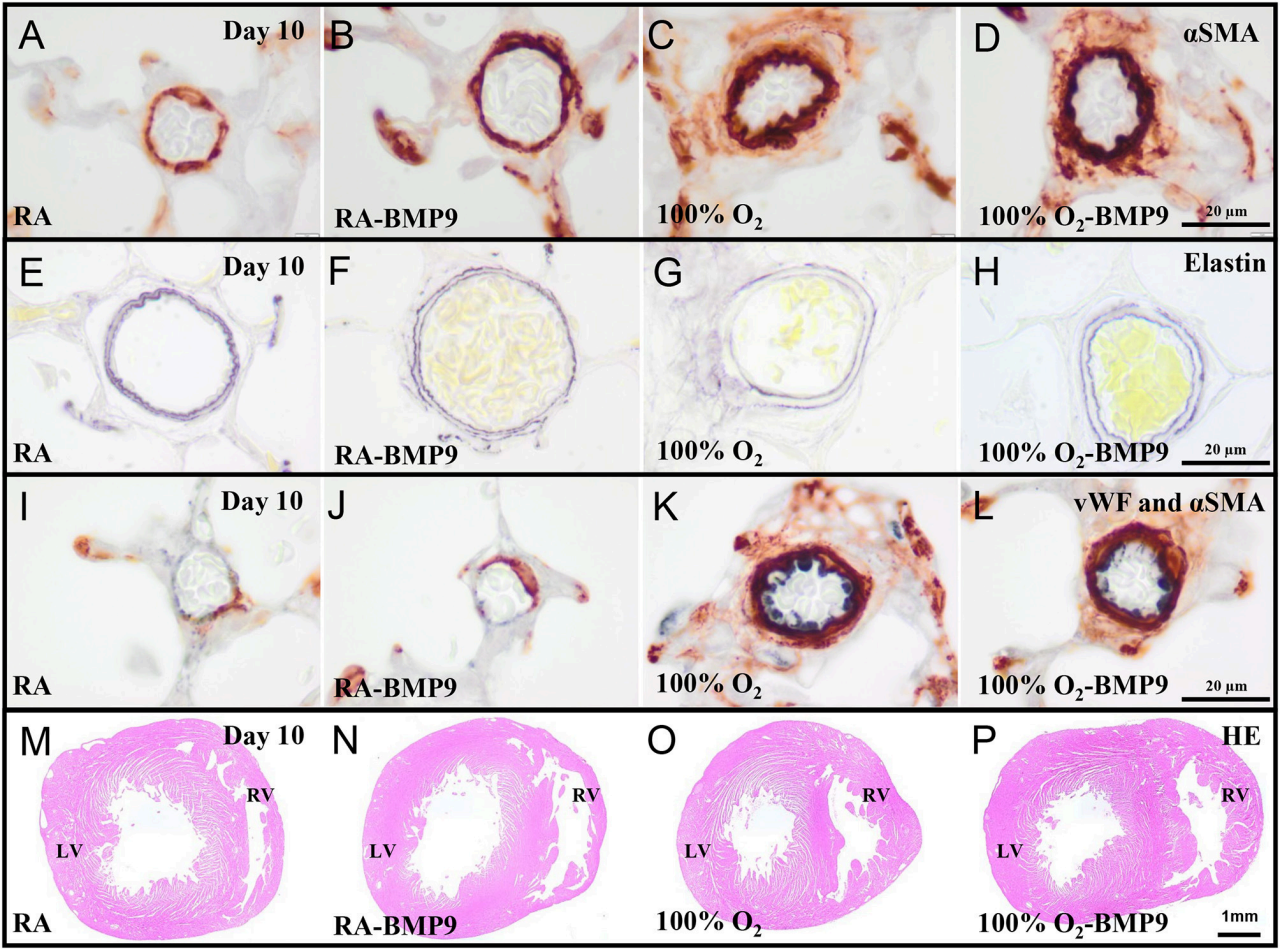
Representative lung sections stained for α smooth muscle actin (αSMA, **A–D**) and elastin **(E–H)**, double-stained for von Willebrand Factor (blue) and αSMA (brown **I–L**) and HE stained heart sections **(M–P)** of rat pups kept in RA **(A,B,E,F,I,J,M,N)** or 100% O_2_ **(C,D,G,H,K,L,O,P)** until 10 days of age. Pups were injected twice a day with 0.9% NaCl or 2.5 μg kg^−1^ BMP9. LV, left ventricle; RV, right ventricle.

**Figure 8:**
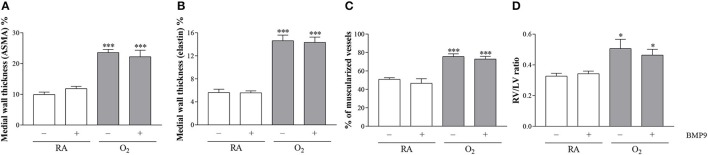
Medial wall thickness was quantified on αSMA-stained lung sections **(A)** and elastin stained sections **(B)**, the percent of muscularized blood vessels was quantified in vWF and αSMA double-stained lung sections **(C)** and the RV/LV free wall thickness ratio was measured in HE-stained heart tissue sections as a marker for right ventricular hypertrophy **(D)** in rat pups kept in RA (open bars) or 100% O_2_ for 10 days (shaded bars). RA and O_2_ pups were injected twice a day for 10 days with 0.9% NaCl or 2.5 μg kg^−1^ BMP9. Values are expressed as mean ± SEM. *N* = 8 for medial wall thickness and vascular muscularization and *N* = 10 for RV/LV ratio. Differences between groups were analyzed by one-way ANOVA, followed by Tukey's multiple comparisons test. ^*^p < 0.05 and ^***^p < 0.001 vs. RA controls. Three independent experiments were performed. LV, left ventricle; RV, right ventricle.

BMP9 did not have adverse effects on pulmonary arterial vascular remodeling (Figures 7B,F,J) and RVH (Figure 7N) during normal neonatal development (Figures 7A,E,I,M). Exposure to 100% O_2_ for 10 days induced vascular remodeling with increased pulmonary arterial medial wall thickness (2.5-fold, *p* < 0.001; Figures 8A,B), determined on αSMA-stained (Figure 7C) and on elastin-stained sections (Figure 7G), and increased muscularization of small arterioles (1.5-fold, *p* < 0.001; Figure 8C), determined on αSMA (brown) and vWF (blue) double stained sections (Figure 7K), as markers for vascular remodeling and PAH. In addition, the ratio RV/LV free wall thickness (1.5-fold, *p* < 0.05; Figures 7O, **8D**) as a marker for RVH increased after exposure to hyperoxia. Treatment with BMP9 for 10 days had no beneficial effect on hyperoxia-induced pulmonary vascular remodeling (Figures 7D,H,L, 8A–C) and RVH (Figures 7P, 8D).

### Effects of BMP9 on pulmonary vascular leakage, extravascular fibrin deposition, IL6 and MCP1 expression, and mRNA expression of ALK1, ALK2, TMEM100, and IL6

**Figure 9:**
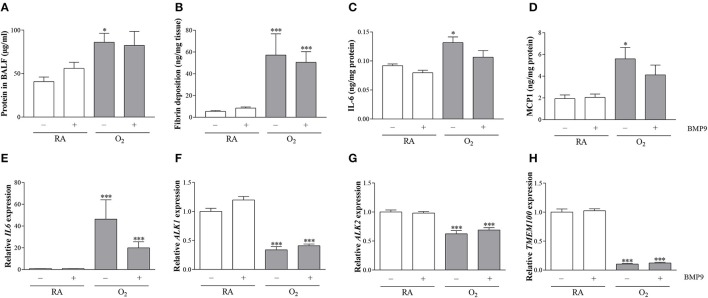
Quantification of total protein concentration in bronchoalveolar lavage fluid (BALF; *N* = 10, **A**) as a marker for vascular leakage, extravascular fibrin deposition (*N* = 8, **B**), protein expression of IL6 (*N* = 8, **C**), and MCP1 (*N* = 8, **D**) and relative mRNA expression (*N* = 8) of *IL6* **(E)**, *ALK1* **(F)**, *ALK2* **(G)**, and *TMEM100* **(H)** in lung tissue homogenates on day 10. RA pups (open bars) and O_2_ pups (shaded bars) were injected twice a day with 0.9% NaCl or 2.5 μg kg^−1^ BMP9 for 10 days. Differences between groups were analyzed by one-way ANOVA, followed by Tukey's multiple comparisons test. Values are expressed as mean ± SEM. ^*^p < 0.05 and ^***^p < 0.001 vs. RA controls.

To establish the effect of pulmonary edema by capillary-alveolar leakage we measured total protein concentration in BALF. Protein concentration on postnatal day 10 was increased 2.1-fold (*p* < 0.05) after exposure to 100% O_2_ compared to RA controls, but was not affected by BMP9 (Figure 9A). Extravascular fibrin deposition in lung tissue homogenates was increased in experimental BPD on neonatal day 10 (10-fold; *p* < 0.001; Figure 9B), but administration of BMP9 did not reduce hyperoxia-induced pulmonary fibrin deposition. IL6 and MCP1 expression in lung tissue homogenates increased 1.4-fold (*p* < 0.05) for IL6 (Figure 9C) and 2.9-fold (*p* < 0.05) for MCP1 (Figure 9D) after exposure to 100% O_2_ for 10 days. Treatment of experimental BPD with BMP9 showed a tendency toward lower IL6 and MCP1 levels. BMP9 had no effect on mRNA expression of *IL6* (Figure 9E), *ALK1* (Figure 9F), *ALK2* (Figure 9G), and *TMEM100* (Figure 9H) during normal neonatal development. Exposure to 100% O_2_ for 10 days increased the expression of *IL6* (42-fold; *p* < 0.001) and decreased the expression of *ALK1* (3-fold; *p* < 0.001), *ALK2* (1.6-fold; *p* < 0.001), and *TMEM100* (10-fold; *p* < 0.001). Treatment with BMP9 for 10 days did not have any effect on the expression of *ALK1, ALK2*, and *TMEM100*, but showed a tendency toward lower *IL6* mRNA levels compared to hyperoxia-exposed controls.

### Binding of BMP9 to its receptor complex activates the intracellular SMAD pathway in endothelial cells

**Figure 10:**
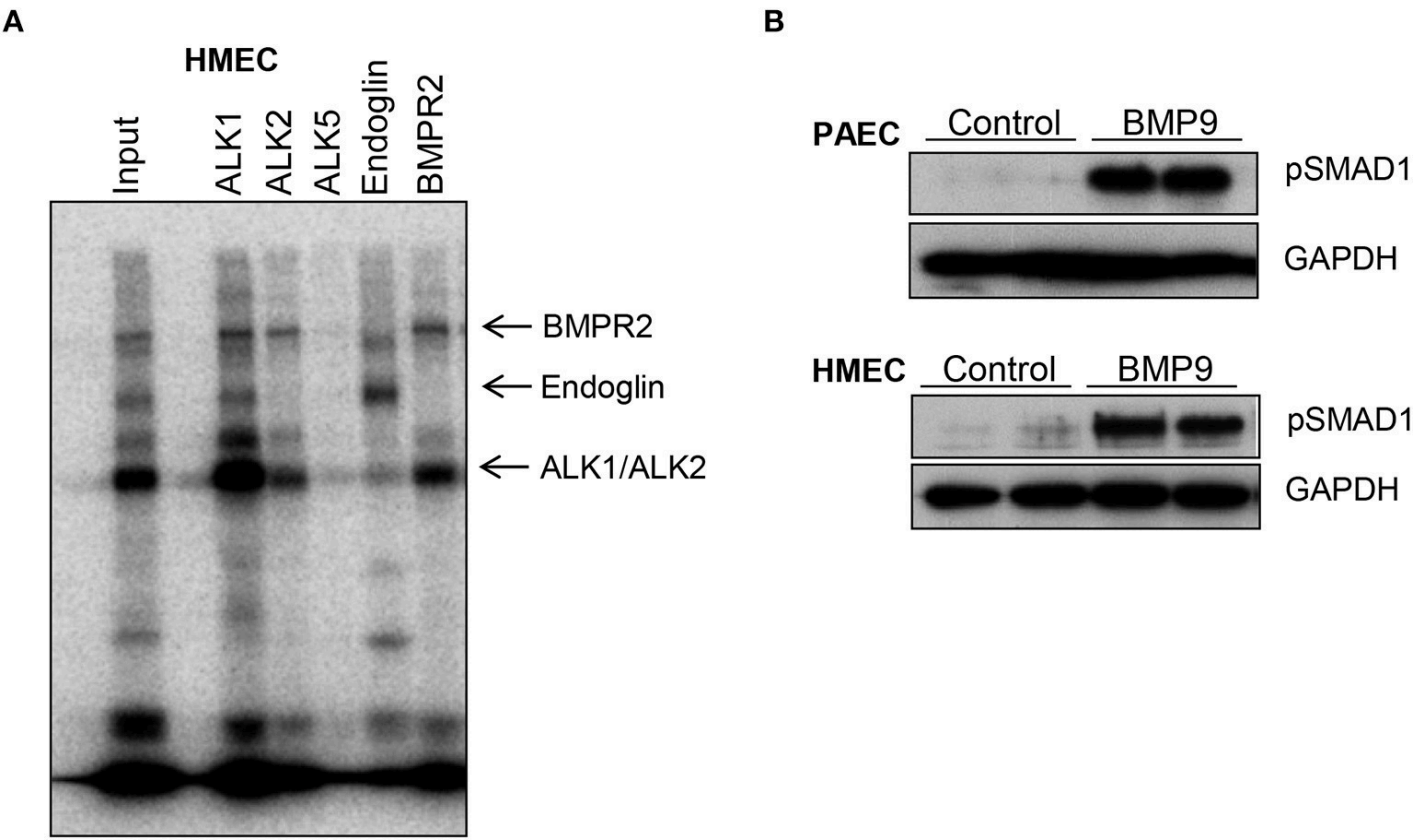
BMP9 binds to endogenous cell surface receptors **(A)** and activates intracellular SMAD pathway in endothelial cells **(B)**. Human microvascular endothelial cells (HMECs) were affinity-labeled with iodinated BMP9 and crosslinked ligand-receptor complexes were immuno-precipitated with specific antisera as indicated **(A)**. BMP9 predominantly binds to ALK1, BMPR2, Endoglin, and weakly to ALK2, but not to ALK5. Input is also shown. Representative Western blots showing BMP9-induced phosphorylated SMAD1 expression in cell lysates of human primary pulmonary arterial endothelial cells (PAEC) and HMEC that were incubated with BMP9 (5 ng/ml) or PBS for 4 h. GAPDH was included as a loading control.

Next we investigated BMP9 binding to its membrane receptor complex in human microvascular endothelial cells (HMEC) after affinity labeling with iodinated BMP9 (Figure 10A) and activation of the intercellular SMAD pathway by BMP9 in endothelial cells (Figure 10B). Crosslinked ligand-receptor complexes were immuno-precipitated with specific antisera against ALK1, ALK2, ALK5, BMPR2, and Endoglin (Figure 10A). BMP9 predominantly binds to ALK1, BMPR2, Endoglin and weakly to ALK2, but not to ALK5. BMP9 activation of the intracellular SMAD pathway was demonstrated by Western blotting in cell lysates of primary human pulmonary arterial endothelial cells (PAEC) and HMEC incubated with BMP9 (5 ng/ml) for 4 h. SMAD1 activation was demonstrated with a phosphorylated SMAD1 specific antibody, using GAPDH as a loading control (Figure 10B).

### BMP9 treatment suppresses endothelial inflammatory cytokine production and stimulates TMEM100 expression *in vitro*

**Figure 11:**
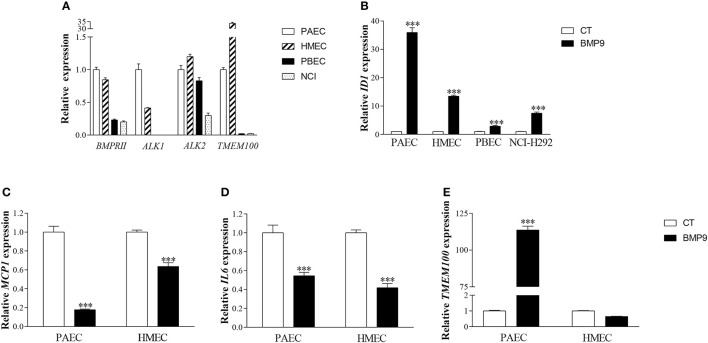
Relative mRNA expression of *BMPRII, ALK1, ALK2*, and *TMEM100* in human primary pulmonary arterial endothelial cells (PAEC), human microvascular endothelial cell line (HMEC), human primary bronchial epithelial cells (PBEC), and human epithelial pulmonary mucoepidermoid carcinoma cell line (NCI-H292) **(A)**. Relative mRNA expression of *inhibitor of DNA binding 1* (*ID1*) in endothelial cells (PAEC and HMEC) and epithelial cells (PBEC and NCI-H292) treated with 5 ng/ml of BMP9 for 4 h **(B)**. Relative mRNA expression of *MCP-1* **(C)**, *IL6* **(D)**, and *TMEM100* **(E)** in endothelial cells (PAEC and HMEC) treated with BMP9 for 4 h. Values are expressed as mean ± SEM. ^***^p < 0.001 vs. Control using *acidic ribosomal protein* (*ARP*) as a reference gene.

The reduction of the pulmonary influx of macrophages and neutrophils, and a trend toward lower IL6 and MCP1 expression in the lungs of BMP9-treated neonatal rats challenged by exposure to hyperoxia suggests that BMP9 administration attenuates the pulmonary oxidative stress-induced inflammatory and fibrotic response. Therefore, we studied the underlying mechanisms by which BMP9 treatment reduces inflammation in the hyperoxia-exposed lung by measuring the expression of important pro-inflammatory cytokines involved in BPD pathology, including *MCP1* and *IL6* (Wagenaar et al., 2004; Bhandari, 2014). First, we analyzed the expression of BMP receptors and the downstream target gene TMEM100 in human primary pulmonary arterial endothelial cells (PAEC), human microvascular endothelial cell line (HMEC), human primary bronchial epithelial cells (PBEC), and the human epithelial pulmonary mucoepidermoid carcinoma cell line NCI-H292 (Figure 11A) and tested their sensitivity to BMP9, by examining the expression of *ID1*, which is a direct target gene of BMP/SMAD signaling (Figure 11B). Both epithelial and endothelial cells expressed BMP receptors *ALK1, ALK2*, and *BMPRII*, and TMEM100 (Figure 11A) and responded to BMP9 with increased expression of *ID1* (Figure 11B). However, epithelial cells showed markedly lower levels of *BMPRII, ALK1*, and *TMEM100* mRNA, which was accompanied with a lower response toward BMP9 stimulation in epithelial cells compared to endothelial cells (Figure 11B). Highest *TMEM100* expression was observed in HMECs (Figure 11A). *ALK2* expression was only reduced in the NCI-H292 cells (Figure 11A). Next we studied the role of BMP9 on pro-inflammatory cytokine production and *TMEM100* expression in endothelial cells (PAEC and HMEC). BMP9 treatment for 4 h significantly decreased *MCP1* (Figure 11C) and *IL6* (Figure 11D) mRNA levels in PAEC and HMEC endothelial cells, and increased *TMEM100* expression in PAECs only (Figure 11E). The absence of BMP9-induced expression of *TMEM100* in HMECs is probably due to its very high expression at basal culturing conditions, compared to PAECs (Figure 11A), thereby preventing additional induction of its expression by BMP9 in HMECs (Figure 11E).

## Discussion

Lung injury induced by chronic exposure to hyperoxia in neonatal rat pups is a valuable *in vivo* model for severe experimental BPD (Wagenaar et al., 2004). BMP9 treatment of rat pups with experimental BPD attenuated neonatal chronic lung disease by improving aberrant alveolar development, demonstrated by reduced alveolar enlargement and septal thickness, and reducing pulmonary inflammation and extravascular collagen deposition. BMP9 did not improve hyperoxia-induced lung aberrant vascularization, pulmonary vascular remodeling, right ventricular hypertrophy (RVH), capillary alveolar leakage and fibrin deposition, nor did it have adverse effects on normal neonatal lung development. The data show that BMP9 may have therapeutic potential to restore aberrant alveolar development and attenuate pulmonary inflammation and fibrosis in preterm infants with severe neonatal chronic lung disease or BPD.

Differential expression of the receptors *BMPRII, ALK1, ALK2*, and *Endoglin*, the ALK1 ligand *BMP9* (David et al., 2007) and the ALK1 downstream gene target TMEM100 (Somekawa et al., 2012) was observed during normal lung development and in hyperoxia-induced neonatal lung disease. Assuming that regulation of gene expression takes place at a transcriptional level, this suggests a role for BMP9/ALK1/BMPRII-dependent signaling in the pathogenesis of severe (experimental) BPD, in which aberrant alveolar and vascular development, vascular remodeling, and inflammation all play a crucial role. Alejandre-Alcazar et al. (2007) reported that mice with hyperoxic neonatal lung injury had higher mRNA expression of TGF-β and BMP superfamily receptors, including ALK1 and BMPRII, compared to RA controls. However, we found a decrease in *ALK1* mRNA expression and an increase in *BMPRII* and *Endoglin* mRNA expression in lungs of hyperoxia-exposed rat pups. This discrepancy in mRNA response to hyperoxia may be explained by differences in species (mice vs. rats), oxygen concentration (85 vs. 100%) and duration of the hyperoxic period (7/14/21/28 vs. 10 days). The role of BMP9 in experimental neonatal chronic lung disease is still unclear. The relatively low expression of *ALK1* and *TMEM100* in hyperoxia-induced neonatal lung injury suggests that BMP9/ALK1/BMPRII-dependent signaling is relatively low in (experimental) BPD. This supports the hypothesis that in hyperoxia-induced neonatal lung disease the balance between BMP- and TGF-β-dependent signaling is disturbed (Hilgendorff et al., 2014) and that restoring this imbalance by either decreasing TGF-β- and/or increasing BMP-dependent signaling may have therapeutic potential. This hypothesis is supported by clinical and experimental data demonstrating that (1) excessive TGF-β-signaling in premature infants and rodents contributes to the development and severity of lung injury and (experimental) BPD (Lecart et al., 2000; Vicencio et al., 2002, 2004; Gauldie et al., 2003; Alejandre-Alcazar et al., 2007; Morty et al., 2009; Tatler and Jenkins, 2012), (2) blocking of TGF-β-signaling reduced lung injury and improved alveolarization in mice with hyperoxia-induced neonatal lung injury (Nakanishi et al., 2007; Sakurai et al., 2011) by preventing the TGF-β-induced downregulation of NO signaling enzymes (Bachiller et al., 2010), and (3) stimulation with BMP9 improves aberrant alveolar development and reduces lung inflammation and fibrosis (this study). Not only does overexpression of TGF-β during early neonatal lung development contribute to experimental BPD, but very low levels of TGF-β activity can result in aberrant lung development with altered cell proliferation, alveolar enlargement, and emphysematous changes in the lung parenchyma (Colarossi et al., 2005), which strongly suggests that TGF-β expression needs to be tightly regulated to preserve normal lung development and prevent lung disease.

The biological role of TMEM100 in lung development and disease is still unclear. In embryonic and fetal lung TMEM100 expression was present in the endothelium of developing pulmonary arteries (Moon et al., 2015). The spatial expression of TMEM100 in the lung could not be confirmed after birth due to the high level of TMEM100 promoter-induced LacZ expression and subsequent X-gal staining in whole mount organ specimens and the absence of proper histological analysis of pulmonary TMEM100 expression after birth (Moon et al., 2010, 2015). TMEM100 knockout mice die in utero on embryonic day 11.5 and lung pathology was only studied in adult inducible knockout mice, showing internal hemorrhages and vascular leakage (Moon et al., 2015). Interestingly, the TMEM100 target gene microfibrillar-associated protein 4 (MFAP4), a protein that is associated with elastin fiber formation, showed a similar pattern of expression in the vasculature of the lung as TMEM100 in our study (Moon et al., 2015). The importance of MFAP4 in postnatal lung development and disease was recently demonstrated in knockout mice, showing spontaneous alveolar enlargement (Holm et al., 2015), strongly suggesting that MFAP4 and TMEM100 are involved in lung development and disease. These data support our findings in experimental BPD, in which aberrant alveolar development was associated with low TMEM100 expression at the mRNA level and by immunohistochemical detection, which is a semi-quantitative measure for protein expression.

The hallmark in BPD is alveolar enlargement which is caused by aberrant alveolar and vascular development (Baraldi and Filippone, 2007). BMP9 attenuated alveolar enlargement and septal thickness in rat pups with hyperoxia-induced neonatal lung injury. Since alveolar development is angiogenesis-driven (Thebaud and Abman, 2007) and pro-angiogenic treatment has therapeutic potential in (experimental) BPD (Thebaud et al., 2005; Balasubramaniam et al., 2006; de Visser et al., 2009, 2010), the beneficial effect on alveolar enlargement may be explained by BMP9/ALK1-dependent stimulation of angiogenesis, which is supported by a reduction in tumor growth in mice and patients by anti-angiogenic therapy with pharmacological blockers of ALK1 (Cunha et al., 2010; Cunha and Pietras, 2011). However, anti-angiogenic properties of BMP9 have also been reported and this effect of BMP9 may vary during lung development and in the adult lung (Long et al., 2015). The absence of a beneficial effect of BMP9 on reduced vascularization suggests that BMP9 improves hyperoxia-induced aberrant alveolarization not by angiogenesis, but by other regulatory mechanisms.

Inflammation plays a pivotal role in the pathophysiology of BPD, because it may contribute to severe lung injury and fibrosis, and treatment with anti-inflammatory agents protects against hyperoxia-induced experimental BPD (Yi et al., 2004; de Visser et al., 2012). BMP9 protected against experimental BPD in rats by attenuating inflammation, as shown by a reduced pulmonary influx of macrophages and neutrophils, less fibrosis, as demonstrated by a reduced extravascular collagen III deposition, and reduced septal thickness. A protective role for BMP9 on lung inflammation in experimental BPD was confirmed by our *in vitro* data. Human endothelial cells expressed the components of the BMP9-ALK1-BMPR2 receptor complex at high levels, showing highest affinity of BMP9 for ALK1, BMPR2, and Endoglin, and after BMP9 stimulation produced high levels of its target genes *ID1*, pSMAD1, and *TMEM100*, thereby confirming the *in vitro* data by Somekawa (Somekawa et al., 2012). BMP9 suppressed the expression of the pro-inflammatory cytokines *IL6* and *MCP1*, suggesting that our control culturing conditions already led to a pro-inflammatory response. Epithelial cells were also sensitive for BMP9, but the expression of *ID1* was much lower, which could be explained by a relatively low level of expression of *ALK1* and *BMPRII* compared to endothelial cells, suggesting a more significant role for the endothelium in the BMP9-dependent suppression of the inflammatory pulmonary response. A protective role of BMP9 on inflammation is supported by *in vitro* and *in vivo* data showing that BMPRII deficiency causes (1) endothelial inflammation, mediated by reactive oxygen species (ROS) and NFκB, and atherosclerosis (Kim et al., 2013), (2) an exaggerated inflammatory response in human and mouse pulmonary artery smooth muscle cells after lipopolysaccharide (LPS) stimulation, which is associated with increased ROS production (Soon et al., 2015), and (3) LPS-induced pulmonary hypertension in mice (Soon et al., 2015). In contrast, a pro-inflammatory role of BMP9 was demonstrated *in vitro* in human endothelial cells and *in vivo* in mice in which BMP9 primed the endothelium, thereby aggravating the acute LPS-induced recruitment of leukocytes (Appleby and Mitrofan, 2016). These data are in sharp contrast to our *in vivo* data in rats in which BMP9 attenuated the inflammatory response. These differences may be explained by disparities in the experimental setup, including differences in the injurious stimulus (LPS vs. oxidative stress) the duration of the injurious stimulus (acute vs. chronic), species (mouse vs. rat), developmental stage (adult vs. neonate), and cell culturing conditions.

We found that 2 × 2.5 μg kg^−1^ day^−1^ of BMP9 effectively improved aberrant alveolar development and prevented the inflammatory response in experimental BPD. This dose of BMP9 is similar to the dosage that most efficiently attenuates PAH in monocrotaline and Sügen-hypoxia-induced PAH in adult rodents (Long et al., 2015). BMP9 did not reduce extravascular fibrin deposition in rat pups with experimental BPD. This was supported by the absence of a beneficial effect on vascular leakage, because extravascular fibrin deposition depends on the leakage of fibrinogen into the alveolar lumen before it can be converted into fibrin by thrombin on damaged lung epithelial cells (Wagenaar et al., 2004).

PAH is a late complication of BPD and associated with right heart disease. Treatment of experimental BPD with BMP9 did not decrease enhanced arteriolar medial wall thickness and muscularization of the small pulmonary arterioles, markers for vascular remodeling and PAH, and RVH in neonatal rat pups. This is in sharp contrast to adult animal models of PAH in which BMP9 and pharmacological activation of BMPR2 with FK506 reduces and even reverses monocrotalin and Sügen-hypoxia-induced PAH and RVH in mice and rats (Spiekerkoetter et al., 2013; Long et al., 2015). This inconsistency in response of BMP9 toward vascular remodeling in neonatal and adult rodents may be explained by the differences in the injurious stimulus and in the onset of lung injury between neonatal and adult rats. In addition, the adult PAH models are characterized by a reduction in lung BMPRII expression (Long et al., 2009), whereas we observed an increase in BMPRII in hyperoxia. Neonatal rats have immature lungs which grow and develop from the saccular to the alveolar stage of development during hyperoxia-induced injury, whereas in adult rodents lung injury is induced in fully mature lungs. BPD is a multifactorial disease in which aberrant alveolarization, inflammation, and vascular remodeling-induced pulmonary hypertension and RVH play an important role. The beneficial effects of BMP9 in experimental BPD are associated with multiple contributing factors and include a major anti-inflammatory and anti-fibrotic effect by preventing the influx of macrophages and neutrophils to the lung, and reducing alveolar enlargement, septal thickness, and collagen III deposition in thick septa. However, BMP9 does not improve survival and prevent vascular remodeling-induced PAH and RVH, which are important late complicating factors of severe (experimental BPD) causing severe morbidity and mortality. Therefore, double treatment with an endothelin receptor inhibitor, which is a potent inhibitor of pulmonary hypertension in the clinic and in experimental BPD (Wagenaar et al., 2013), may be considered in addition to BMP9 to improve treatment of severe (experimental) BPD.

The differential expression of *ALK1, ALK2, and TMEM100* in rat pups with experimental BPD was not accompanied by a significant down regulation of phosphorylated SMAD1 and SMAD2, and was not affected by BMP9 treatment. These data suggest that BMP9-ALK1-SMAD1-dependent signaling in the pulmonary vasculature and/or lung epithelium may at least in part be mediated via protein kinase activation rather than via SMAD-dependent BMP9 signaling (Pardali and Ten Dijke, 2012). In addition, our *in vitro* studies using human endothelial cells suggest that in the absence of the high affinity receptor ALK1 BMP9-dependent signaling may occur via binding of BMP9 to ALK2.

Aberrant osteogenesis is a potential adverse effect of BMP9 that may hamper the translation of BMP9 to the clinic (Kang et al., 2004). However, we did not observe adverse effects in rat pups treated with BMP9 for 10 days and in adult rats that were treated for PAH with the same concentration of BMP9 for a period of 3 weeks (Long et al., 2015), demonstrating that BMP9-induced aberrant osteogenesis in our neonatal and adult rat models is absent. If negative effects of treatment with BMP9 in human infants are absent, extrapolation of the beneficial effects of BMP9 treatment in rat pups with experimental neonatal chronic lung disease to preterm infants with respiratory failure may result in a novel therapeutic treatment option for preterm infants with severe BPD by improving aberrant alveolar development and reducing pulmonary inflammation and fibrosis, which are major contributors to mortality and morbidity.

## Author contributions

FW, GF, PH, PtD, MG, NM, and GW participated in research design. XC, MO, EL, AH, and AH-E conducted experiments. XC, MO, EL, AH, and GW performed data analysis. XC, MO, FW, PtD, NM, and GW wrote or contributed to the manuscript.

### Conflict of interest statement

The authors declare that the research was conducted in the absence of any commercial or financial relationships that could be construed as a potential conflict of interest.
